# Genetic variants of *CHRNA5-A3* and *CHRNB3-A6* predict survival of patients with advanced non-small cell lung cancer

**DOI:** 10.18632/oncotarget.8510

**Published:** 2016-03-30

**Authors:** Yang Wang, Xiaonu Peng, Lijun Zhu, Likuan Hu, Yipeng Song

**Affiliations:** ^1^ Department of Radiation Oncology, Yantai Yuhuangding Hospital, Yantai, Shandong, China; ^2^ Department of Oral and Maxillofacial Surgery, Guangdong General Hospital and Guangdong Academy of Medical Sciences, Guangzhou, China; ^3^ Department of Radiation Oncology, Qilu Hospital of Shandong University, Jinan, Shandong, China

**Keywords:** CHRNA5-A3, CHRNB3-A6, survival, nicotinic acetylcholine receptors, lung cancer

## Abstract

Nicotinic acetylcholine receptors (nAChRs) play a key role in carcinogenesis and progression of lung cancer; and polymorphisms in *CHRNA5-A3* and *CHRNB3-A6*, two gene clusters encoding nAChR subunits, have been associated with lung cancer risk. In this study, we investigated whether variants in the two gene clusters were associated with prognosis of advanced non-small cell lung cancer (NSCLC). A total of 165 stage IIIB–IV NSCLC patients were enrolled in this study. Three polymorphisms (rs667282 and rs3743073 in *CHRNA5-A3* and rs13280604 in *CHRNB3-A6*) were genotyped using the TaqMan method. Overall survival (OS) was estimated using the log-rank test and the Cox models. Our results showed that patients with *CHRNA5-A3* rs667282 TT or TC genotypes had a significantly shorter OS than those carrying the CC genotype (Log-rank, *P* = 0.043). Furthermore, multivariate Cox regression analysis showed that rs667282 TT/TC genotypes are significantly associated with increased risk of overall deaths (adjusted hazard ratio, 1.7; 95% CI, 1.1–2.7). However, the similar results were not observed for other two polymorphisms. Furthermore, no evident association was found between these variants and clinicopathologic features of advanced NSCLC. Our present study suggested that rs667282 in *CHRNA5-A3* may modify the prognosis of patients with advanced NSCLC.

## INTRODUCTION

Lung cancer is the most common cause of cancer-related deaths worldwide. Approximately 1.6 million lung cancer cases and 1.4 million lung cancer deaths occurred in 2015 globally [1]. Non-small cell lung cancer (NSCLC) accounts for about 80% of primary lung cancers, with most of the patients diagnosed at the advanced stage (stage III or IV) [2]. Despite advances in chemotherapy and radiotherapy, prognosis of advanced NSCLC patients is still unimproved with a 5-year survival rates less than 15% [3]. Historically, treatment and prediction of prognosis for these patients is usually based on clinical characteristics, such as smoking behaviors, performance status (PS) and tumor, lymph node and metastasis (TNM) staging system [4–6]. New biomarkers that allow the identification of NSCLC patients with high risk of death/recurrence will facilitate the development of specific, less toxic, more aggressive for selected, targeted treatment strategies to improve therapeutic efficacy and outcomes. Thus, identification of such genetic prognostic biomarkers is needed.

Nicotinic acetylcholine receptors (nAChRs) are ion channels that are universally expressed in the plasma membrane of all mammalian cells, including cancer cells [7]. Now nAChRs have been identified as central regulators of stimulatory and inhibitory neurotransmitters that govern the synthesis and release of growth, angiogenic and neurotrophic factors in cancer cells and cancer microenvironment [8]. Recently, several single nucleotide polymorphisms (SNPs) in two nAChR subunit-encoding gene clusters (rs1051730, rs16969968 and rs8034191 in *CHRNA5-A3* and rs13280604 in *CHRNB3-A6*) were shown to be associated with lung cancer risk and smoking consumption in Caucasians by genome-wide association (GWA) studies [9–12]. However, the three SNPs (rs1051730, rs16969968, and rs8034191) of *CHRNA5-A3* reported in GWA studies are extremely rare in Chinese population, according to the HapMap database and a study of Wu et al. [13], while two independent studies found that rs667282 and rs3743073 in *CHRNA5-A3* affected lung cancer risk in Chinese population [13, 14]. On the other hand, in addition to rs13280604 there are several other SNPs, including rs6474412, rs1451240, rs10958725, rs4736835, rs10958726, rs4950, and rs6474415 in *CHRNB3-A6* region, while these SNPs are either in strong linkage disequilibrium (LD) or even perfect LD (*R*^2^ > 0.8) with rs13280604. Moreover, the association of rs13280604 in *CHRNB3-A6* with smoking consumption and lung cancer risk has been observed in multiple ethnic populations, including Korean from East Asia [15]. However, to date no studies have evaluated if these variants in the two gene clusters also contribute to the prognosis of lung cancer, particularly the advanced NSCLC. Consequently, we investigated the associations of these three SNPs (rs667282, rs3743073 and rs13280604) in *CHRNA5-A3* and *CHRNB3-A6* with survival of advanced NSCLC patients.

## RESULTS

### Patient characteristics

The median age of the patients was 61 years (aged 30–82). There were 134 males (81.2%), 99 ever-smokers (60%) and 140 patients (84.8%) having a performance status of 0 or 1. For the histological types, 87 (52.7%) patients were diagnosed with adenocarcinoma, 60 (36.4%) with squamous cell carcinoma and 18 (10.9%) with other types. The majority of the patients (69.1%) presented with stage IV NSCLC. A total of 106 patients received conventional, platinum-based chemotherapy, 26 patients received radiotherapy, 22 patients received a combination of radiotherapy and chemotherapy, and 11 patients received treatment by other methods. Demographic and clinical characteristics of patients are summarized in Table 1.

**Table 1:** Demographic and clinical characteristics of NSCLC patients

Characteristics	*N* (%)
Median age (range)	61 (30–82)
≤ 60	81 (49.1)
> 60	84 (50.9)
Gender	
Male	134 (81.2)
Female	31 (18.8)
Smoking status	
Ever	99 (60)
Never	66 (40)
ECOG PS^a^	
0–1	140 (84.8)
2	25 (15.2)
Histological type	
Squamous cell carcinoma	60 (36.4)
Adenocarcinoma	87 (52.7)
Others	18 (10.9)
Clinical stage	
IIIB	51 (30.9)
IV	114 (69.1)
Treatment	
Chemotherapy	106 (64.2)
Radiotherapy	26 (15.8)
Chemoradiotherapy	22 (13.3)
Others	11 (6.7)

a ECOG PS, Eastern Cooperative Oncology Group performance status.

### Associations of genotypes with clinicopathological characteristics of patients

All patients had the genotyping data for analysis, and the genotype distributions of patients are presented in Table 2. We found that there were no significant differences in distribution of genotypes of these polymorphisms in term of clinicopathological features, such as age, gender, smoking status, EGOG performance status, histology, stage, and treatment (*P* > 0.05, Table 2).

**Table 2:** Genotype distribution of three polymorphisms by clinicopathological characteristics

	rs667282			rs3743073			rs13280604		
Characteristics	CC *n* = 30	TT/TC *n* = 135	P*	TT *n* = 44	GG/TG *n* = 121	P*	AA *n* = 92	GG/AG *n* = 73	P*
Age (years)									
≤ 60	11	70	0.13	23	58	0.62	43	38	0.50
> 60	19	65		21	63		49	35	
Gender									
Male	24	110	0.85	37	97	0.57	73	61	0.49
Female	6	25		7	24		19	12	
Smoking status									
Never	12	54	1.00	16	50	0.56	35	31	0.56
Ever	18	81		28	71		57	42	
ECOG PS^a^									
0–1	26	114	0.76	36	104	0.51	79	61	0.68
2	4	21		8	17		13	12	
Histological type									
Adenocarcinoma	16	71	0.94	27	60	0.18	48	39	0.87
Others	14	64		17	61		44	34	
Clinical stage									
IIIB	8	43	0.58	12	39	0.54	33	18	0.12
IV	22	92		32	82		59	55	
Treatment									
Chemotherapy	25	103	0.40	35	93	0.72	67	61	0.10
Others	5	32		9	28		25	12	

a ECOG PS, Eastern Cooperative Oncology Group performance status.

* *P* values were calculated by the χ^2^ tests.

### Associations of genotypes with overall survival

**Figure 1:**
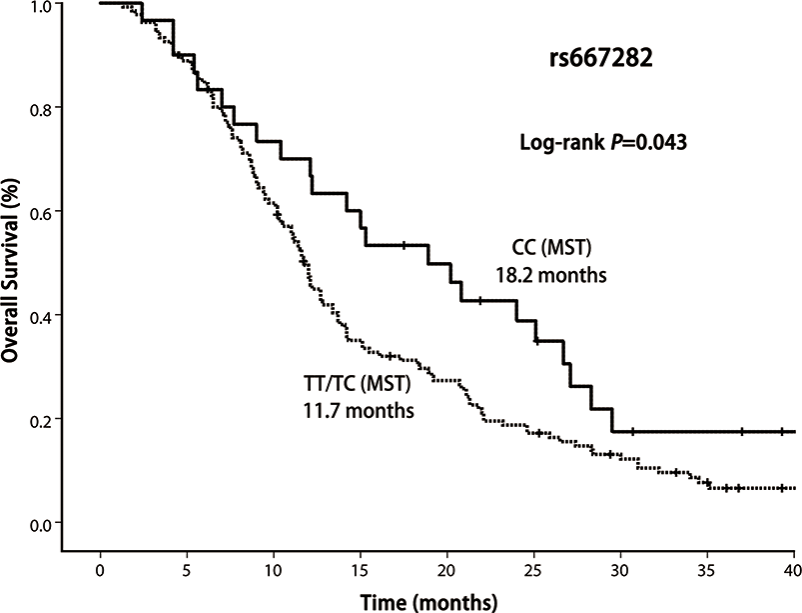
Kaplan–Meier curve of OS according to CHRNA5-A3 rs667282 polymorphism genotypes

**Figure 2:**
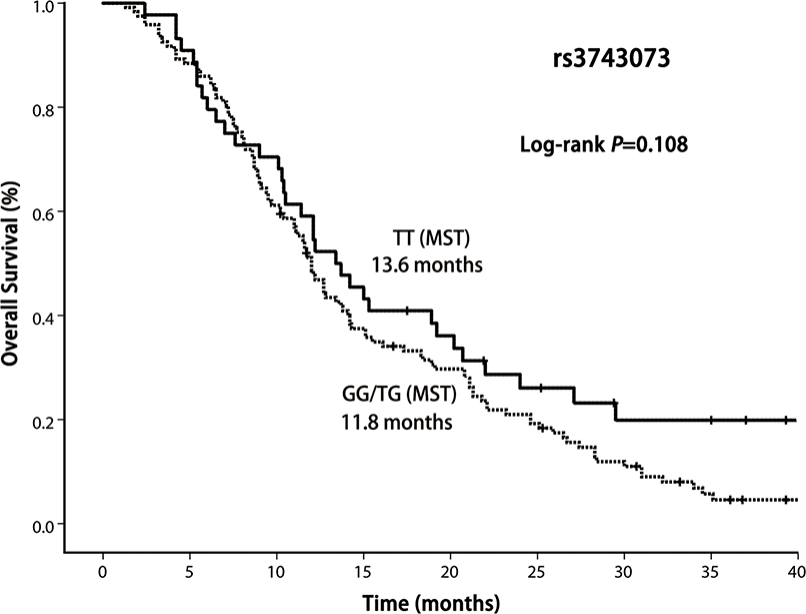
Kaplan–Meier curve of OS according to CHRNA5-A3 rs3743073 polymorphism genotypes

**Figure 3:**
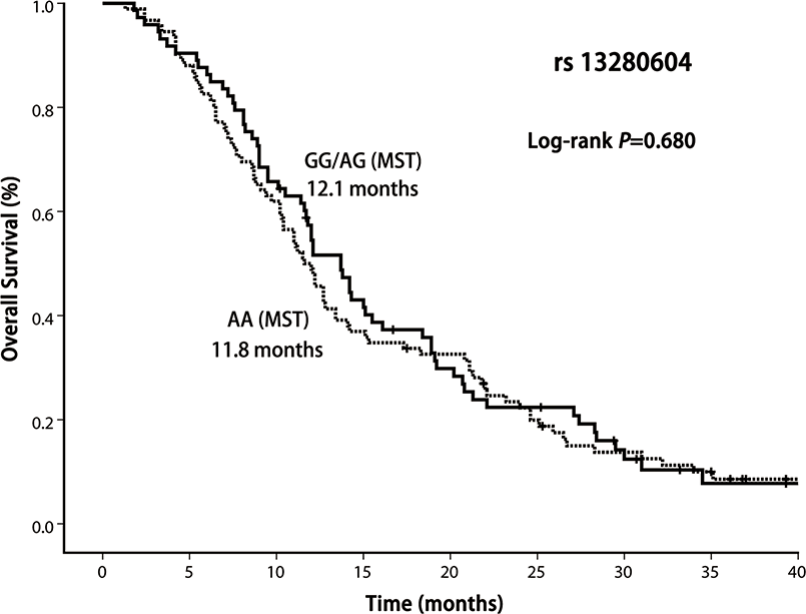
Kaplan–Meier curve of OS according to CHRNB3-A6 rs13280604 polymorphism genotypes

In this study, 13 patients were alive and 7 patients were lost to follow-up. In the entire group, the median survival time was 12.1 months (range 1.3–41.7 months). Our result demonstrated that OS was significantly shorter among the patients with *CHRNA5-A*3 rs667282 TT/TC genotypes compared with those carrying the CC genotype (Log-rank *P* = 0.043, Figure 1), while the similar significant differences in OS was not observed for *CHRNA5-A3* rs3743073 (log-rank *P* = 0.108, Figure 2) and *CHRNB3-A6* rs13280604 polymorphisms (log-rank, *P* = 0.680, Figure 3), respectively. Multivariate Cox regression analysis was used to assess the associations of three polymorphisms with full adjustment of other confounding factors including age, gender, smoking status, ECOG PS, histological type, clinical stage, and treatment, with risk of overall deaths (Table 3). The results showed that rs667282 TT/TC genotypes were significantly associated with increased risk of overall deaths among patients with advanced NSCLC (HR, 1.7, 95% CI, 1.1–2.7), independent of other prognostic factors. However, the significant associations were not found between the genotypes of other two polymorphisms and risk of overall deaths (data not shown).

**Table 3:** Multivariable analysis on association between risk of overall deaths and rs667282 genotypes in advanced NSCLC patients

Variables	HR*	95% CI	*P*
Age (years)			
≤ 60			
> 60	1.1	0.8–1.6	0.53
Gender			
Male			
Female	0.8	0.5–1.2	0.29
Smoking status			
Never			
Ever	1.6	1.1–2.3	0.02
ECOG PS^a^			
0–1			
2	1.7	1.1–2.7	0.03
Histological type			
Adenocarcinoma			
Others	1.3	0.9–1.8	0.21
Clinical stage			
IIIB			
IV	1.3	0.9–1.9	0.15
Treatment			
Chemotherapy			
Others	1.1	0.7–1.6	0.78
rs667282			
CC			
TT/TC	1.7	1.1–2.7	0.02

* adjusted by age, gender, smoking status, ECOG PS, histological type, clinical stage, and treatment.

## DISCUSSION

In the current study, we examined whether the SNPs in the two gene-clusters, which are significantly associated with lung cancer risk, are also associated with prognosis of lung cancer. According to the analysis of 165 advanced NSCLC patients, we found that rs667282 in *CHRNA5-A3* was significantly associated with OS in NSCLC patients. Furthermore, multivariate Cox regression analysis showed that this SNP was an independent indicator for NSCLC prognosis after adjusting for confounding factors, including age, gender, smoking status, ECOG PS, histological type, clinical stage and treatment. No significant association was observed between the other two polymorphisms and survival.

nAChRs are the important regulators of cancer development and progression owing to their high affinity for tobacco-specific carcinogenic nitrosamines 4-(methylnitrosamino)-1-(3-pyridyl)-1-butanone (NNK) and N-nitrosonornicotine (NNN) [8]. In addition, nAChR subunit-encoding gene cluster *CHRNA5-A3* may have function in chemotherapy resistance to gemcitabine, cisplatin, and paclitaxel in NSCLC through the Akt-dependent proliferation and the NF-kappaB-dependent survival pathways under the stimulation of NNK [16–18]. Previous studies reported that rs667282 TT/CT genotypes in *CHRNA5-A3* have been shown to be associated with increased smoking consumption which is always linked with a poor prognosis of lung cancer [13, 19], which is biologically consistent with our finding in the current study.

Although many studies have focused on the impacts of nAChR subunit-encoding genes on lung cancer risk and smoking consumption, few reports have been reported regarding the associations between the polymorphisms in these genes with survival outcomes. Recently, Jin et al. examined the association of a polymorphism rs6495309 in *CHRNA5-A3*, which was in strong linkage disequilibrium with rs667282 [13], with prognosis in patients with early-stage, surgically resected NSCLC [20]. They found that the polymorphism was significantly associated with survival outcome, consistent with the finding in our study in advanced NSCLC patients. In addition, there exists a risk tendency for another polymorphism rs3743073 in *CHRNA5-A3*, while such trend was not statistically significant. Niu's et al. found [14] that rs3743073 was associated with lung cancer risk, however, no evidence in an association with smoking consumption, while found for rs667282, was observed.

To our knowledge, this is the first study that investigated the association between polymorphisms in *CHRNB3-A6* and lung cancer prognosis. The polymorphism rs13280604 was first reported to be associated with increased smoking consumption in the study of Saccone et al. [21]. They analyzed 3713 SNPs of 348 candidate genes in 1050 nicotine dependent cases and 879 controls, and observed a significant effect of the SNP on smoking behaviors in population of European descents. Subsequently, this association with smoking has been reported in multiple other ethnic populations, including African-American, European-American and Korean [15, 22–24]. Recently, a GWA study revealed that the variant in *CHRNB3-A6* was significantly associated with both smoking and lung cancer risk in population of European descents; however, the effect of rs13280604 on smoking consumption was much weaker than those of the polymorphisms in *CHRNA5-A3* [9]. It might implicate that the effect of rs13280604 on lung cancer prognosis may be independent of its interference with smoking consumption. In contrast, no association was found between this polymorphism, which has been found to be associated with lung cancer risk, and NSCLC survival of in the current study. Thus, further investigation in effect of this polymorphism in *CHRNB3-A6* on lung cancer prognosis is warranted.

Several limitations need to be addressed in this study. Because of the limited sample sizes of NSCLC patients, results from this study may be biased. Thus future studies with a larger number of NSCLC cases are expected to further confirm our findings. Moreover, a relatively longer follow-up period is needed to investigate such associations between these variants in *CHRNA5-A3* and *CHRNB3-A6* with lung cancer survival outcome. In addition, in the study, we had no detailed information on smoking duration and intensity, which were required to calculate smoking pack-year. This limits our ability to assess the involvement of smoking level in the association. Finally, the majority of the study patients are diagnosed at late stage, and such analyses in clinical outcome with inclusion of early stage patients are needed in our future studies. In conclusion, this study first reveals that rs667282 in *CHRNA5-A3* is significantly associated with survival outcome of NSCLC patients, indicating that the SNP may serve as a useful genetic biomarker for the prognosis of advanced NSCLC patients. However, additional large well-designed studies with different ethnic populations are needed to confirm these findings, particularly in other smoking-related cancers, such as head and neck cancers and esophageal squamous cell carcinoma in Chinese population.

## MATERIALS AND METHODS

### Study patients and follow-up

The subjects in this study consisted of a total of 165 patients with cytologically or histologically confirmed stage IIIB–IV NSCLC from Qilu Hospital of Shandong University between May 2009 and June 2010. We staged patients according to the 7th edition of the TNM classification of the International Union Against Cancer (UICC). Eligibility criteria included age ≥ 18 years, Eastern Cooperative Oncology Group (ECOG) performance status ≤ 2, and adequate organ function. All patients were unrelated ethnic Chinese who were all from Northern China. In this study, the primary endpoint for survival analysis was overall death. NSCLC patients were typically followed and monitored throughout their treatment and post-treatment courses with regularly scheduled clinical and radiographic examinations. Medical record review for the follow-up statuses of all patients was performed under the direct supervision of the surgeon. Overall survival (OS) was defined as the time from initial diagnosis until death from any cause or last follow-up. Participants who were alive at the end of the study period or lost to follow-up were censored. The study was performed in accordance with the Helsinki Declaration, and was approved by the Ethics Committees of Qilu Hospital of Shandong University. At recruitment, all participants were required to give written informed consent.

### Genotyping

Whole-blood samples were collected from all study participants at the time of study entry. We extracted genomic DNA from the peripheral blood samples using the Tiangen Biotech kit (DP319; Tiangen Biotech (Beijing) Co., Ltd., Beijing China). The rs667282 and rs3743073 polymorphisms in *CHRNA5-A3* and the rs13280604 polymorphism in *CHRNB3-A6* were genotyped by TaqMan allelic discrimination technology, which uses two allele-specific TaqMan MGB probes and a PCR primer pair to detect the specific SNP target. Primers and probe information are shown in Table 4. PCR amplifications were conducted in a 96-well Applied Biosystems 7500 Real Time PCR System according to the instructions of the manufacturer and SDS 1.4 software (Applied Biosystems) was used for allelic discrimination.

**Table 4:** Primers and probes used for the TaqMan genotyping

Polymorphism	Sequence (5′-3′)
*CHRNA5-A3* rs667282	
Primer	F: TGGACTTTTCTACAACCTTGCTACTT
	R: GCCTGAGACTCTGCATTTCTAACAA
Probe	FAM: ACAGTATTCAC*G*TCACCTG
	VIC: ACAGTATTCAC*A*TCACCTG
*CHRNA5-A3* rs3743073	
Primer	F: GGAGAAGGAGACGGTAAAAGAATCA
	R: TGTGGTTCGGTCACATGCA
Probe	FAM: TGGTTTTACTTCCC*T*TGGCACC
	VIC: TTTTACTTCCC*G*TGGCACC
*CHRNB3-A6* rs13280604	
Primer	F: GTGCTCCCTGTGAAGGTACA
	R: GCTTGCTGCCCCTGGAT
Probe	FAM: AATCCTGCTC*T*TCACCAAG
	VIC: CCTGCTC*C*TCACCAAG

Ten percent of the samples were randomly chosen for re-testing, and the results were in 100% concordance with those of the initial assays.

### Statistical analyses

Associations between genotypes of the three SNPs and individual clinicopathologic factors were assessed using the Pearson chi-square (χ^2^) test. OS curves were drawn with the Kaplan–Meier product limit method for each of the different genotype groups. Comparisons were tested with the log-rank test. Hazard ratios (HRs) and 95% confidence intervals (CIs) of risk genotypes were estimated by fitting the Cox model while adjusting for age, sex, smoking status, performance status, histological type, clinical stage and treatment regimen. Statistical analyses were performed with the SPSS software package (version 20). *P* values of less than 0.05 were taken to indicate statistically significant differences, and all of the statistical tests were two-sided.

## Abbreviations

nAChRs: nicotinic acetylcholine receptors
NSCLC: non-small cell lung cancer
OS: overall survival
SNP: single nucleotide polymorphisms
HR: Hazard ratios
CI: confidence intervals
